# Intelligently chosen interventions have potential to outperform the diode bridge in power conditioning

**DOI:** 10.1038/s41598-019-45103-4

**Published:** 2019-06-20

**Authors:** Feiyang Liu, Yulong Zhang, Oscar Dahlsten, Fei Wang

**Affiliations:** 1 Physics, Southern University of Science and Technology (SUSTech), Shenzhen, China; 2 School of Microelectronics, Southern University of Science and Technology (SUSTech), Nanshan District, Shenzhen, China; 3 Shenzhen Institute for Quantum Science and Engineering, (SUSTech), Nanshan District, Shenzhen, China; 4 London Institute for Mathematical Sciences, Mayfair, 35a South Street, London, W1K 2XF UK; 5 Wolfson College, University of Oxford, Oxford, OX2 6UD UK

**Keywords:** Devices for energy harvesting, Electronic and spintronic devices

## Abstract

We probe the potential for intelligent intervention to enhance the power output of energy harvesters. We investigate general principles and a case study: a bi-resonant piezo electric harvester. We consider intelligent interventions via pre-programmed reversible energy-conserving operations. These include voltage bias flips and voltage phase shifts. These can be used to rectify voltages and to remove destructive interference. We choose the intervention type based on past data, using machine learning techniques. We find that in important parameter regimes the resulting interventions can outperform diode-based intervention, which in contrast has a fundamental minimum power dissipation bound.

## Introduction

Energy harvesting, exploiting ambient energy for our purposes, plays a crucial role in human technological development^1^. Currently, an important focal area is micro energy harvesters (with output power 10–100 *μ*W). These convert, through various transduction methods, ambient thermal and kinetic energy from the environment to electrical energy. They provide an *in-situ* power source for remote electronic devices, typically for powering sensor nodes of the Internet of Things. This avoids the problems associated with batteries and/or wiring^2–6^.

A key challenge for micro harvesters is that ambient energy sources are very often random. For instance, the amplitude and the frequency of a vibrational energy source can be highly variable. This makes it difficult to rectify the generated voltage/current and store the energy in an efficient manner^2,3,7^.

Interventions by an intelligent agent aids energy harvesting from variable sources in certain contexts, as exemplified by the interventions of a sailor, or a windvane turning a generator into the wind. Such examples serve to remind us that the 2nd law of thermodynamics, as used to prove that a Maxwell’s demon cannot work^8^, concerns maximum entropy single heat baths, whereas often forces in nature are not maximally random.

Highly sophisticated intelligent interventions in energy harvesting are now practicable, owing to advances in: (i) artificial intelligence software and hardware^9,10^, (ii) electronic interfacing circuitry^11–15^, and (iii) experimental and theoretical understanding of the relation between information and energy, such as the fact that reversible computation has no fundamental energy cost^16–18^. Taken together, this gives significant hope that intelligent intervention may be a powerful tool in mitigating the randomness faced by micro-harvesters.

We therefore here aim to identify intelligent interventions that allow micro harvesters to extract more rectified power from variable sources than current state-of-the art methods.

**Figure 1:**
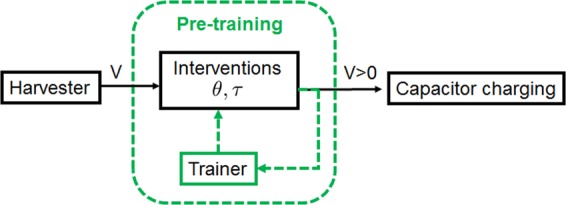
We find that rectifying the voltage with intelligently chosen pre-programmed energy conserving interventions can lead to improved power output. Possible interventions include voltage bias flip with ON/OFF-period *τ*, and voltage phase shift of time *θ*. The trainer tunes *θ* and *τ* to optimise the voltage output.

A key paradigm we adapt is to use interventions that are reversible and energy conserving. Moreover, for practical and fundamental thermodynamical reasons, these interventions are pre-programmed, chosen by systematic machine learning methods applied to past data from the harvester, as in Fig. 1. We consider general principles and for concreteness also a case study of a piezo-electric harvester which converts motion to electricity^19^. In this case study we simulate idealised, pre-programmed, bias-flips and phase-shifts on experimental output data. We find these can indeed replace and outperform the current state-of-the art: the diode bridge.

We moreover note that the the diode bridge has a thermodynamically fundamental lower bound on power dissipation, whereas the methods use here do not.

We proceed as follows. We briefly describe the harvester being used as a case study. We describe the interventions, and how they can be intelligently chosen. We then give the results, followed by a discussion and conclusion.

## Methods

**Figure 2:**
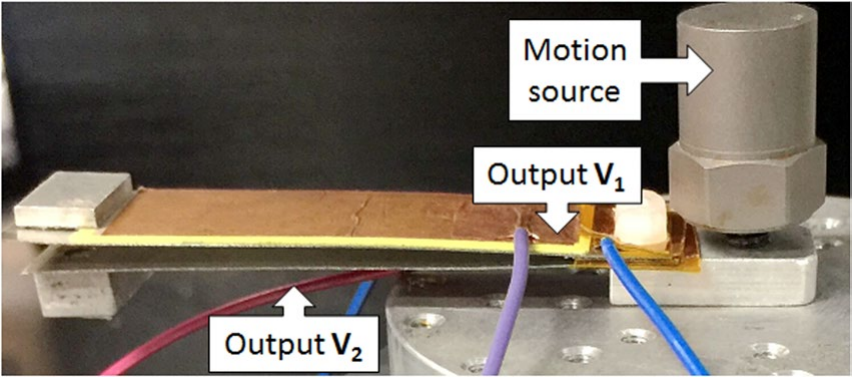
The bi-resonant harvester, originally designed in^19^. Two piezo-covered cantilevers with masses on their free ends are driven by the same vibrational motion source on the right axis. We consider how much bias flips and phase shifts on the outputs can enhance the power output.

The harvester we use in this paper as a case study is a dual resonant structure energy harvester^19^, which can harvest energy from random fluctuation sources at low frequencies (typically less than 100 Hz), consistent with motion of everyday objects such as human beings. As shown in Fig. 2, it consists of two piezoelectric devices, each outputting its own voltage time-series, with the voltages finally combined to give one voltage time-series. Both the two cantilevers are made of stainless steel with dimensions of 67 (length) 18 (width) 0.2 mm (thickness). On the surface of each cantilever, there is glued a lead zirconate titanate (PZT-5A) film with dimensions of 52 (length) 17 (width) 0.2 mm (thickness). The measurement is performed when the two piezoelectric devices are mounted on a shaker (Brüel&Kjær, 4810) to mimic the vibration sources from ambient environment.

We have used a signal generator (Brüel&Kjær, LAN-XI 3160) and a power amplifier (Brüel&Kjær, 2719), to generate excitation signals with tunable frequency and amplitude. An accelerometer is mounted with the energy harvesters to monitor the real time vibrations. The signal from the piezoelectric devices is connected to a data acquisition unit. It should be noted that even though piezoelectric devices are used for proof of the concept, the proposed method could also be useful for other related energy harvesters. We also remark that since the parasitic capacitance is low relative to load resistance, and the vibration is at low frequency, we may approximate the current and voltage to be fully in phase. Thus the instantaneous power respects $P=\frac{{V}^{2}}{R}$ for a load resistance *R* and the average power respects $\langle P\rangle =\frac{{V}_{{\rm{RMS}}}^{2}}{R}.$

### Diode bridge and its power consumption

**Figure 3:**
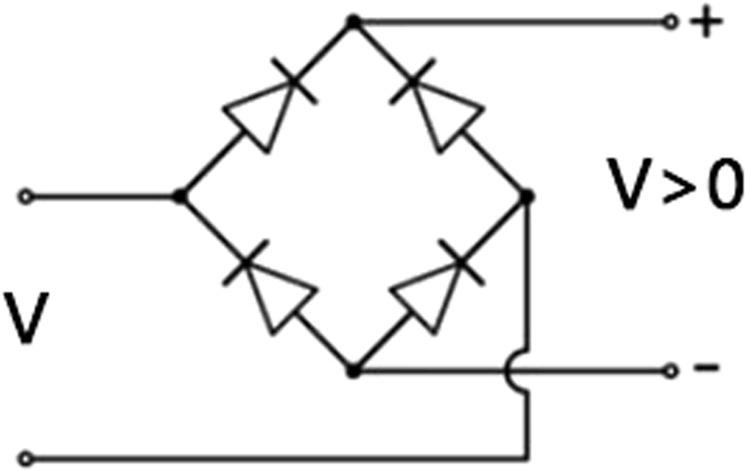
A diode bridge will take any voltage polarity on the inputs to a positive polarity on the output.

A diode bridge, as in Fig. 3, will take any voltage polarity on the inputs to a positive polarity on the output, but at a loss in power.

The loss in power is necessary given that each diode has a voltage drop. For practical device power dissipation calculations a pn-junction diode’s current vs voltage curve can be approximated as *V* = *V*_0_ + *IR* for the regime *V* > 0^20^. The instantaneous power dissipated by a single diode when *V* > 0 is then *P* = *IV*_0_ + *I*^2^*R*. (The average and rms power dissipations follow immediately). The diode bridge has two diodes in each path and thus twice that dissipation.

Note also that in the case of two sub-harvesters the diode bridge can be applied on each before combining the voltages in order to avoid destructive interference, but again at a power loss.

### 2nd law mandates diode bridge power consumption

If it were possible to reduce the above power dissipation to 0, a diode bridge could be used to violate the second law of thermodynamics, by turning thermal current fluctuations into rectified current at no work cost. Kelvin’s version of the 2nd law states that no work can be extracted from a single heat bath in a closed cycle. Thermal voltage fluctuations depend on materials and it is beyond the scope of this paper to investigate their values for diode bridges used here, but e.g. the thermal voltage *V*_*th*_ = *kT*/*e* is approx 0.03 V at room temperature. (The argument can be modified to other fluctuation sizes).

For the device to be called a diode the current *I* ≈ 0 for negative voltages beyond the thermal fluctuation range of −*V*_*th*_ (up to some breakdown voltage which is outside of the range currently considered). Then for voltages in the positive thermal fluctuation range we must also have *I* ≈ 0, or else the diode would generate a current in a circuit embedded in the heat bath, a circuit which could include a load driven by that current, violating the 2nd law. Thus, according to the above argument, there is an inescapable voltage drop of *V*_*th*_ in a diode, and associated power loss. We now turn to the competing approach to turn the AC into DC and to remove destructive interference between voltages.

### Two examples of intelligent interventions: sign flip and phase shift

The sign flip, which can also be called voltage inversion, can be written as *V* → −*V* where *V* is the instantaneous voltage. This can switch between being on and off with period *τ*. *τ* is a priori a free parameter and will later be set according to optimising based on past data.

The phase shift is simply a delay of the voltage time series by some amount *ϕ* (so strictly speaking it is a delay rather than a phase shift which should only be between 0 and 2 *π* times some period). It can be written as *V*(*t*) → *V*(*t* + *ϕ*) ∀ *t* where *t* is time.

### Interventions are orthogonal matrices

It is convenient to use bra-ket vector notation here such that a voltage time series *V*_*i*_(*t*_0_), ... *V*_*i*_(*t*_*f*_) is a vector |*V*_*i*_〉 with the first entry *V*_*i*_(*t*_0_). (The time series is discrete as it is sampled experimentally at a finite rate). The transpose of the vector is denoted 〈*V*_*i*_|, such that the dot product of two vectors |*V*_*i*_〉, |*V*_*j*_〉 is written as 〈*V*_*i*_||*V*_*j*_〉 = 〈*V*_*i*_|*V*_*j*_〉. In this notation

1 $${V}_{{\rm{RMS}}}=\sqrt{\frac{1}{d}{\langle {V}_{i}|V\rangle }_{i}},$$

where *d* is the dimension of |*V*_*i*_〉. Moreover let $|{V}_{i}^{^{\prime} }\rangle$ denote the transformed |*V*_*i*_〉. This means the voltage time series one would get instead of |*V*_*i*_〉 if the intelligent transformations are implemented.

In an idealised case the intelligent transformations preserve *V*_RMS_ such that

2 $${\langle {V}_{i}|V\rangle }_{i}=\langle {V^{\prime} }_{i}|{V^{\prime} }_{i}\rangle \forall i\mathrm{.}$$

Then, if we also assume the interventions can be represented as matrices, the interventions correspond to orthogonal matrices *O*, meaning *O*^*T*^*O* = *I* where *I* is the identity and *T* the transpose. The idealised interventions we consider are indeed orthogonal matrices: phase shifting can be represented as a cyclic permutation of elements, a particular permutation matrix, and voltage inversion as a diagonal matrix with diagonal entries all 1 or −1. More generally the interventions ${\mathscr{S}}$ are naturally represented as matrices, since they should respect probabilistic mixtures of different voltages: ${\mathscr{S}}({\sum }_{i}{p}_{i}|{V}_{i}\rangle )={\sum }_{i}{p}_{i}{\mathscr{S}}(|{V}_{i}\rangle )$ ^21^. This together with Eq. 2 implies the idealised interventions, beyond the examples of bias flips and phase shifts, should indeed be represented as orthogonal matrices acting on the voltage vectors.

### Optimal interventions when combining two voltages

Now we can compare the *V*_RMS_ before and after interventions. For example phase shifts can be used to reduce destructive interference, due to individual sub-generators producing voltages out of phase. Given two or more voltage time series, how high can the *V*_RMS_ of the combined outputs be, if we are allowed to do intelligent interventions on each time series before combining them? For notational convenience let us consider $d{V}_{{\rm{RMS}}}^{2}=\langle V|V\rangle$. Two time series illustrate the general case: *V*_1_ and *V*_2_. Suppose these undergo the transforms before being combined, how much can $d{V}_{{\rm{RMS}}}^{2}$ change? Note that

3 $$\begin{array}{c}\langle {V^{\prime} }_{1}+{V^{\prime} }_{2}|{V^{\prime} }_{1}+{V^{\prime} }_{2}\rangle -\langle {V}_{1}+{V}_{2}|{V}_{1}+{V}_{2}\rangle =\,\mathrm{2(}\langle {V^{\prime} }_{1}|{V^{\prime} }_{2}\rangle -\langle {V}_{1}|{V}_{2}\rangle \mathrm{).}\end{array}$$

Thus maximising the *V*_RMS_ improvement for a given |*V*_1_〉, |*V*_2_〉 means maximising $\langle {V^{\prime} }_{1}|{V^{\prime} }_{2}\rangle$. Can we find a closed form expression for how high $\langle {V^{\prime} }_{1}|{V^{\prime} }_{2}\rangle$ can be? Let us consider maximising it over permutation matrices and sign flips. Note firstly that making the signs the same for all entries, e.g. plus, cannot decrease $\langle {V^{\prime} }_{1}|{V^{\prime} }_{2}\rangle$. We can assume that in the optimal case the signs are the same, say all positive. Now it is known that the dot product is maximised by ordering the entries of each in descending order: $\langle {V^{\prime} }_{1}\downarrow |V^{\prime} {\downarrow }_{2}\rangle$. This follows from the rearrangement inequality^22^. Thus the maximum $d{V}_{{\rm{RMS}}}^{2}$ one can obtain by signflips and permutations is

4 $$\begin{array}{c}ma{x}_{{\rm{sgnflip}}+{\rm{perms}}}\langle {V^{\prime} }_{1}+{V^{\prime} }_{2}|{V^{\prime} }_{1}+{V^{\prime} }_{2}\rangle \\ \,=\,\langle {V}_{1}|{V}_{1}\rangle +\langle {V}_{2}|{V}_{2}\rangle +2\langle {V}_{1}\downarrow |{V}_{2}\downarrow \rangle :\,=d{V}_{{\rm{RMS}}}^{(max\mathrm{)2}}\mathrm{.}\end{array}$$

Physically, phase shifts, which correspond to a restricted set of permutations (cyclic permutations) appear significantly more realistic than arbitrary permutations. We shall therefore in this paper consider phase shifts and sign-flips. This means that the optimum derived in Eq. 4 cannot necessarily be obtained, but one can hope to approach it.

### Energy cost of these interventions arbitrarily small

If transformations take individual microstates to other microstates with the same energy they can in principle be performed without an energy cost. Moreover there needs to be a one-to-one mapping between microstates for there not to be a hidden energy cost owing to thermodynamics^16^. For example compressing a gas to half its volume isothermally does not change the (average) internal energy of the gas but nevertheless costs work, associated with the reduction of the entropy.

The interventions here, in idealised form, satisfy those conditions, whereas the diodes do not. The bias flip does not change the potential energy associated with the voltage difference. The phase shift is a delay, again not changing the potential energy. Moreover both operations are reversible, as can be seen physically, and from the fact that orthogonal matrices are reversible.

In contrast, the diode bridge is logically and thermodynamically irreversible with an inescapable lower power dissipation, as discussed above.

We are investigating what the energetic cost of the interventions will be in practise, taking hope from e.g.^11^ that it can be made low enough to be practical. We also remark here that the interventions are similar to a feedforward quantum neural net^23^ wherein the transformations are also reversible (unitary), providing another possible physical platform for these ideas.

### Cost function used for the training

We wish to optimise the *V*_RMS_ of the output, under the restriction that it should be DC, i.e. *V* > 0. This latter condition is because typically small energy harvesters need to produce DC, e.g. to charge a capacitor.

For a single voltage the cost function quantifying how far we are from only having positive voltage (POS) can be conveniently implemented as

5 $${C}_{POS}=4\langle |V|||V|\rangle -\langle \tilde{V}|\tilde{V}\rangle ,$$

where $|\tilde{V}\rangle =||V|\rangle +|V\rangle \mathrm{.}$ One sees that if all entries are positive, *C* = 0, and otherwise *C* > 0.

Moreover we define

6 $${C}_{{V}_{{\rm{RMS}}}}=d{V}_{{\rm{RMS}}}^{{({\rm{\max }})}^{2}}-d{{V}_{{\rm{RMS}}}}^{2},$$

where ${V}_{{\rm{RMS}}}^{({\rm{\max }})}$ is the maximal over intelligent interventions of Eq. 4. For simplicity we define the total cost function, taking both desired properties into account, as

7 $$C={C}_{{V}_{{\rm{RMS}}}}+{C}_{POS}\mathrm{.}$$

A protocol we shall investigate here is where the phase shift is done on two individual voltages before combining them, followed by a joint sign flip. In this case we accordingly use the cost function

8 $$\begin{array}{rcl}C(\tau ,\varphi ) & = & {C}_{{V}_{{\rm{RMS}}}}+{C}_{POS}\\  & = & [\langle {V}_{1}\downarrow ||{V}_{2}\downarrow \rangle \,-\,\langle {V^{\prime} }_{1}|{V^{\prime} }_{2}\rangle ]+[4\langle |V^{\prime} ||V^{\prime} |\rangle -\langle \tilde{V^{\prime} }||\tilde{V^{\prime} }\rangle ],\end{array}$$

where |*V*′〉 is the sum of the two voltages after the first phase shift and $|\tilde{V^{\prime} }\rangle =||V^{\prime} |\rangle +|V^{\prime} \rangle .$

### Systematic training methods exist

To have a systematic and scalable approach we consider the well-proven machine learning/optimisation technique of gradient descent on a suitably defined cost function. The gradient descent rule as applied here is that

9 $$(\begin{array}{c}\tau \\ \theta \end{array})\to (\begin{array}{c}\tau \\ \theta \end{array})-\eta (\begin{array}{c}\frac{C(\theta ,\tau +\delta )-C(\theta ,\tau )}{\delta }\\ \frac{C(\theta +\delta ,\tau )-C(\theta ,\tau )}{\delta }\end{array})$$

where *δ* and *η* are numerical parameters chosen according to what works.

Moreover, when faced with local minima in the cost function landscape, we employ the genetic algorithm, a type of evolutionary algorithm commonly used to find global minima when there are many local minima. In the genetic algorithm, the global minimum (highest fitness generation) can often be found, after operations like mutation, crossover and selection^24^.

The experimental data used was 16384 voltage readings taken at a very high frequency (8194 Hz) relative to the generator frequency (29 Hz). We used some of the time-series data (80%) for determining the optimal interventions, and then tested those interventions on the remaining data (20%).

The training used here can be classified as *reinforcement learning*, as the performance is evaluated (rather than the output being compared to a a known correct answer as in supervised learning).

### Simulated intelligent intervention beats diode bridge

Our simulation shows that a combination of periodic voltage inversion and phase shift provides DC voltage that is higher than that after the diode bridge.

The diode bridge penalty of about 0.2 V is significant in regimes where *V*_RMS_ is of the order of 0.2 or less. It is in these regimes it makes sense to consider replacing the diode bridge.

**Figure 4:**
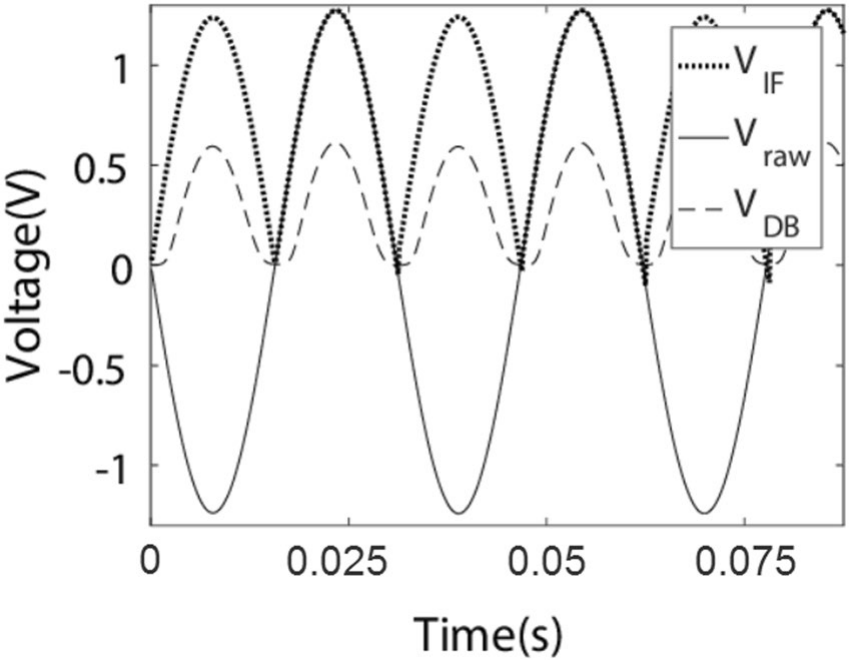
Intelligently chosen periodic voltage inversion gives better *V*_RMS_ performance than the diode bridge for the same input. The diode bridge data is fully experimental. The intelligent intervention data is from applying the corresponding orthogonal matrix on the raw data experimental data before the diode bridge. *V*_*IF*_ is the voltage after the intelligently chosen flip (experiment + simulated intervention), *V*_RAW_ (experimental) is the direct output from the harvester, and *V*_DB_ is that after the diode bridge (experimental).

Figure 4 shows how the *V*_RMS_ is left essentially undiminished and approximately non-negative by an intelligently chosen periodic voltage inversion, whereas the diode bridge loses about half of the *V*_RMS_. In regimes of even lower *V*_RMS_ this advantage will be even greater of course.

**Figure 5:**
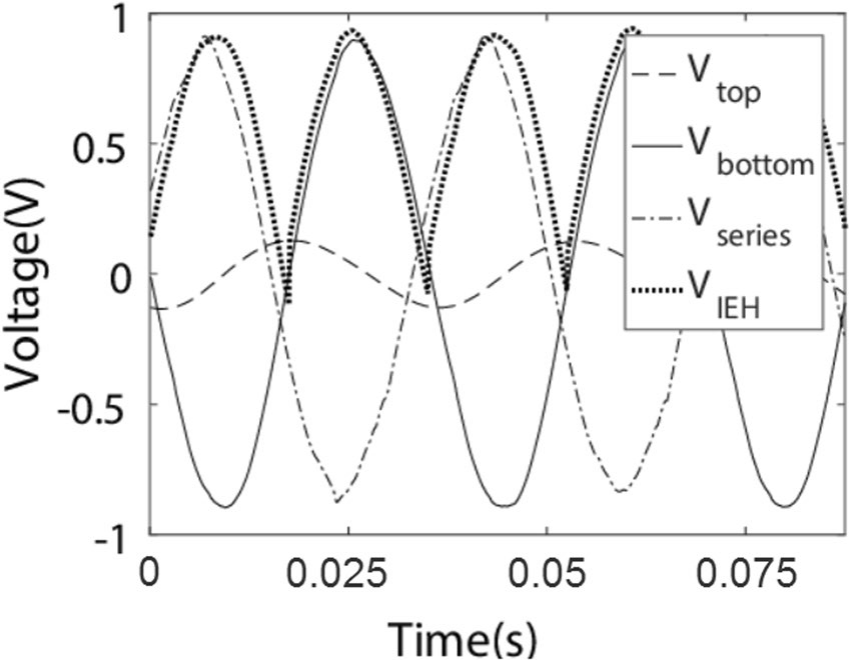
Two devices driven by the same source can have very similar frequency but be out of phase, as in this experimental data based on 2 piezo-electric harvesters. The phase difference leads to negative interference. The output of intelligent energy harvesting reduces the consumption of voltage and maximizes the RMS.

**Figure 6:**
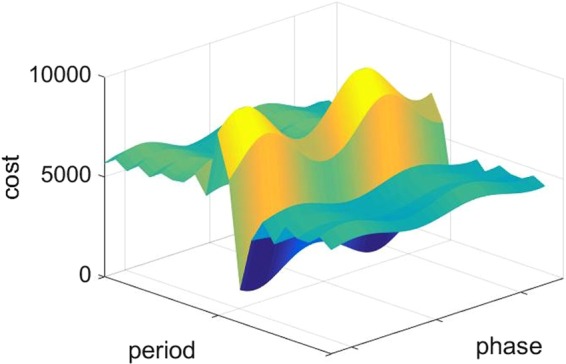
Cost function landscape. X-axis is period and Y-axis is delay(length of phase shift), Z-axis is the cost function value, which combines a cost for being less than 0 and a cost for having suboptimal Vrms.

In the case of two sub-generators we find that the simulated intelligently chosen delay plus intelligently chosen periodic inversion can also in principle significantly outperform the diode bridge, as shown in Fig. 5. An example of the cost function landscape for real experimental data combined with simulated intervention is in Fig. 6, showing local minima, which is why we employed the genetic algorithm for the training. The *V*_RMS_ improvement here is given in Table 1.

**Table 1:** Comparison of different methods in terms of *V*_RMS_ and DC character of output achieved.

${{\boldsymbol{V}}}_{{\bf{R}}{\bf{M}}{\bf{S}}}(\frac{{{\boldsymbol{C}}}_{{\boldsymbol{P}}{\boldsymbol{O}}{\boldsymbol{S}}}}{{\boldsymbol{L}}})$	1 voltage	2 voltages
raw data	0.89 (1.6)	0.59 (0.7)
DB	0.37 (0.5)	0.22 (0.2)
IEH	0.89 (0.01)	0.59 (0.47)

Three methods are considered: (i) No intelligent intervention or diode bridge (NO DB, NO IEH), (ii) diode bridge is applied (DB), (iii) intelligent interventions are applied instead (IEH). 2 scenarios are included: (a) a 1-voltage output harvester, and (b) 2-voltage output harvester. In (a) & (IEH) only the periodic bias flip is employed. In (b) & (IEH) there is a phase shift applied, followed by combining the voltages, followed by a periodic bias flip. For each scenario method the *V*_RMS_ is displayed with a measure of the DC character of the output in brackets, corresponding to *C*_*POS*_/*L* where *C*_*POS*_ is defined in Eq. 5 and *L* is the length of the time series.

### Signal-to-noise ratio important

We consider adjusting the power of additive Gaussian white noise to see the change of *V*_RMS_ and *C* of the intelligent interventions and and diode bridge respectively.

**Figure 7:**
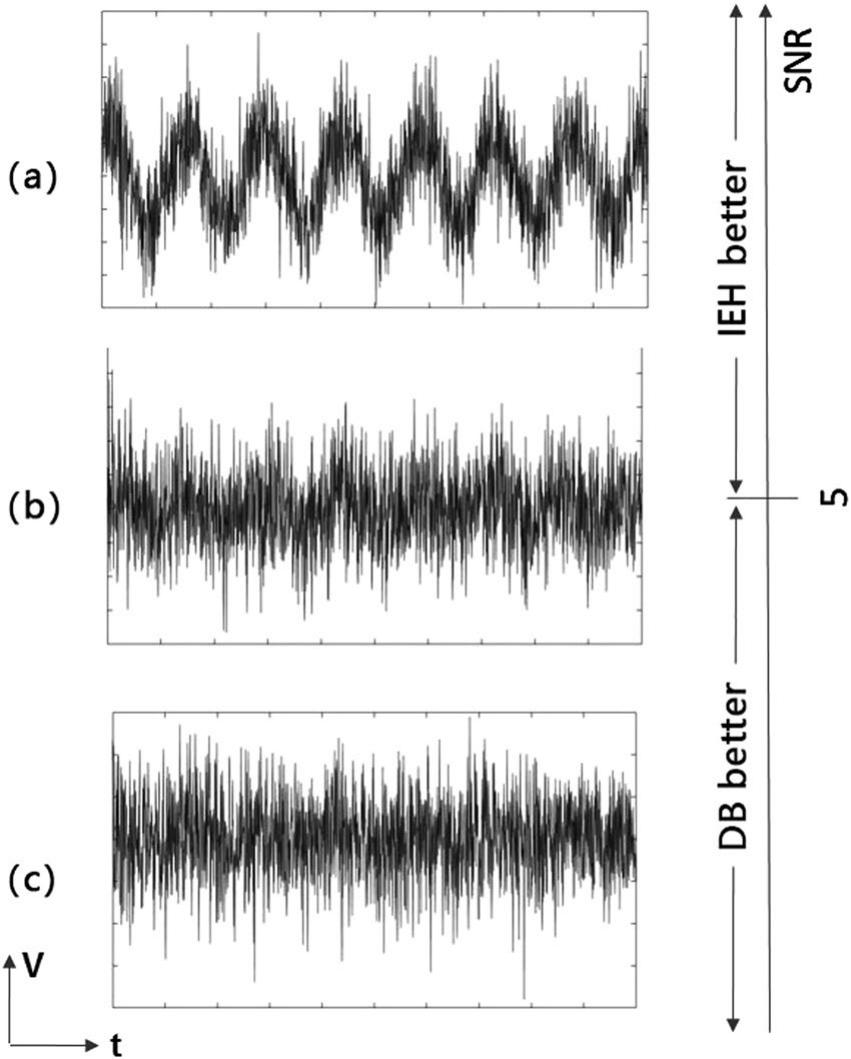
Comparison of diode bridge and intelligent intervention under noise. The signal voltage series are raw experimental data and the noise is added numerically. There are three cases illustrating the corresponding parameter regime: (**a**) SNR(signal-to-noise ratio) is greater than 5, for which we find that the intelligent harvesting (IEH) has a better cost function performance, (**b**) SNR is close to 5, wherein the cost of IEH and the diode bridge (DB) are similar, and (**c**) SNR is less than 5, wherein we find the cost of the diode bridge is lower.

We find that whilst the *V*_RMS_ of the intelligent interventions is always greater than the diode bridge, when it comes to the total cost function *C*, it looks like in Fig. 7: there is a threshold signal-to-noise ratio SNR after which the diode bridge wins. This is consistent with the understanding that the intelligent intervention relies on patterns existing.

## Conclusion

We have simulated interventions on experimental voltage data to investigate whether they could outperform the diode bridge in terms of DC voltage output. We find that in the regime of good signal-to-noise ratio and low voltages this is indeed the case. A reason the low-voltage regime is important is the continuing miniaturisation of e.g. piezo and tribo-electric harvesters. We conjecture that many natural scenarios give rise to good signal-to-noise ratio. This naturally gives rise to follow-on questions: (i) will experimental implementations of the interventions perform comparably to our idealised simulations? (ii) what natural power sources and harvesters do/do not give rise to voltages with good signal-to-noise ratios?

## Supplementary information

Supplementary

Supplementary Dataset 1

Supplementary Dataset 2

Supplementary Dataset 3

Supplementary File - source code

## Data Availability

The datasets generated during and analysed during the current study are available from the corresponding author on reasonable request.

## References

[1] Coopersmith, J. *Energy, the subtle concept: the discovery of Feynman’s blocks from Leibniz to Einstein*. (Oxford University Press Oxford 2010).

[2] Mitcheson PD, Yeatman EM, Rao GK, Holmes AS, Green TC (2008). Energy harvesting from human and machine motion for wireless electronic devices. Proceedings of the IEEE.

[3] Kong, L. B. *et al. Waste Energy Harvesting: Mechanical and Thermal Energies*. (Springer 2014).

[4] Sudevalayam S, Kulkarni P (2011). Energy harvesting sensor nodes: Survey and implications. IEEE Communications Surveys & Tutorials.

[5] Zhang Y (2018). Micro electrostatic energy harvester with both broad bandwidth and high normalized power density. Applied Energy.

[6] Zhang Y, Yushen H, Xinge G, Wang F (2018). Micro energy harvester with dual electrets on sandwich structure optimized by air damping control for wireless sensor network application. IEEE Access.

[7] Halvorsen E (2008). Energy harvesters driven by broadband random vibrations. Journal of Microelectromechanical Systems.

[8] Bennett Charles H. (1987). Demons, Engines and the Second Law. Scientific American.

[9] Linares-Barranco B, Andreou AG, Indiveri G, Shibata T (2003). Guest editorial - special issue on neural networks hardware implementations. IEEE Transactions on Neural Networks.

[10] LeCun Yann, Bengio Yoshua, Hinton Geoffrey (2015). Deep learning. Nature.

[11] Ramadass Y, Chandrakasan A (2010). An efficient piezoelectric energy harvesting interface circuit using a biasflip rectifier and shared inductor. Solid-State Circuits, IEEE Journal Of.

[12] Bandyopadhyay S, Chandrakasan AP (2012). Platform architecture for solar, thermal, and vibration energy combining with mppt and single inductor. IEEE Journal of Solid-State Circuits.

[13] Liang J, Chung HS-H (2013). Best voltage bias-flipping strategy towards maximum piezoelectric power generation. Journal of Physics: Conference Series.

[14] Kim J, Kim C (2013). A dc–dc boost converter with variation-tolerant mppt technique and efficient zcs circuit for thermoelectric energy harvesting applications. IEEE Transactions on Power Electronics.

[15] Hartmann F, Pfeffer P, Höfling S, Kamp M, Worschech L (2015). Voltage fluctuation to current converter with coulomb-coupled quantum dots. Phys. Rev. Lett..

[16] Bennett CH (1982). The thermodynamics of computation, a review. IJTP.

[17] Sagawa, T. Second law-like inequalities with quantum relative entropy: An introduction. In *Lectures on Quantum Computing, Thermodynamics and Statistical Physics*, 125–190 (World Scientific, 2013).

[18] Goold J, Huber M, Riera A, del Rio L, Skrzypczyk P (2016). The role of quantum information in thermodynamicsa topical review. Journal of Physics A: Mathematical and Theoretical.

[19] Li S, Peng Z, Zhang A, Wang F (2016). Dual resonant structure for energy harvesting from random vibration sources at low frequency. AIP Advances.

[20] ST SN604 Application note, w. Calculation of power loss in a diode (2011).

[21] Barrett J (2007). Information processing in generalized probabilistic theories. Phys. Rev. A.

[22] Hardy, G., Littlewood, J. & Plya, G. *Inequalities, Cambridge Mathematical Library (2. ed.)* (Cambridge University Press 1952).

[23] Wan, K. H., Dahlsten, O., Kristjánsson, H., Gardner, R. & Kim, M. S. Quantum generalisation of feedforward neural networks. *npj Quantum Information* **3**, 36, 1612.01045 (2017).

[24] Holland, J. H. Adaptation in natural and artificial systems: An introductory analysis with applications to biology, control, and artificial intelligence. http://mathworks.com/discovery/genetic-algorithm.html (1975).

