# Supplementary material for: Intelligently chosen interventions have potential to outperform the diode bridge in power conditioning
Source: Sci Rep. 2019 Jun 20;9:8994. doi: 10.1038/s41598-019-45103-4 (PMC6586793; doi:10.1038/s41598-019-45103-4)
Supplement: Supplementary file 1 — Supplementary [file 41598_2019_45103_MOESM1_ESM.pdf]

7 May 2019

Supplementary material for *Intelligently chosen interventions have potential to outperform the diode bridge in power conditioning.*

Feiyang Liu [1], Yulong Zhang (co-first)[2], Oscar Dahlsten [1, 3, 4, 5], and Fei Wang (co-corr.)[2],

1 *Physics, Southern University of Science and Technology (SUSTech), Shenzhen, China*

2 *EEE, Southern University of Science and Technology (SUSTech), Nanshan District, Shenzhen, China*

3 *Shenzhen Institute for Quantum Science and Engineering, SUSTech, Nanshan District, Shenzhen, China*

4 *London Institute for Mathematical Sciences, Mayfair, 35a South Street, London, W1K 2XF, UK*

5 *Wolfson College, University of Oxford, Oxford OX2 6UD, UK*

The supplementary material contains 7 files in addition to this readme file.

4 files are matlab code for the training as well as plotting. The main code (main.m) calls two functions related to the cost function: costfun_calc.m calculates the cost function and costfun_graph.m calculates it in a way convenient for plotting on a mesh grid. vf.m is a function called by both cost function files, and is used to generate voltage flips for a given ON/OFF period.

The other three files are voltage time series data from experiment, used in the paper. The files are labelled according to voltage activating shaker, the frequency of the sampling and the resistance used (M means megaohms).

```
---------------------------------------------------
filename main.m
---------------------------------------------------

clear
clc

%%%%%%%%%%input data%%%%%%%%
global data1 data2
data1 = textread('V_top device_200mv_29Hz_0.04762M.txt');
data2 = textread('V_bottom device_200mv_29Hz_0.04762M.txt');
```

```
V1 = data1(:,3)';
V2 = data2(:,3)';
L = length(V1);
t = 1:1:floor(L);
V1r = V1;
V2r = V2;



%%%%%%%%%make landscape%%%%%%%%
tao1 = 1:1:floor( 0.01*L);
tao2 = 1:1:floor(  0.04*L);
for i = 1:1:floor( 0.01*L)
   for j = 0:1:floor( 0.04*L)-1
      cost(i,j+1)= costfun_graph(V1,V2,i,j);
   end
end
[tao1,tao2] = meshgrid(tao1,tao2);
mesh(tao1,tao2,cost')


%%%%%%%%training by genetic algorithm%%%%%%%%%
V1 = V1r(1:1:floor(0.2*L));
V2 = V2r(1:1:floor(0.2*L));
Vdb = V1+V2;
Vdb = max(abs(Vdb)-0.5,0);
x = ga(@costfun_calc,2,[],[],[],[],[1 1],[200 200]);
V1 = circshift(V1,floor(x(2)));
V2 = circshift(V2,-73);
L = length(V1);
flip = vf(x(1),L);
Vtot = V1+V2;
Vtot = Vtot.*flip;   %%%%%%final output voltage%%%%%

---------------------------------------------------
filename costfun_calc.m
---------------------------------------------------

function cost = costfun_calc(tau)
% Calculates the cost function. Tau contains both inversion period
and phase shift.
global data1 data2


%import the two voltage time series
V1 = data1(:,3)';
V2 = data2(:,3)';
```

```
%split off a part for training
L = length(V1);
V1 = V1(1:1:floor(0.2*L));
V2 = V2(1:1:floor(0.2*L));


%apply phase shift
V1 = circshift(V1,floor(tau(2)));
L = length(V1);

%generate a vector with plus and minus ones corresponding to flips,
%with on-off period given by tau(1).
flip = vf(floor(tau(1)),L);
%apply the flip to the voltages added in series
Vtot = V1+V2;
Vtot = Vtot.*flip;

%evaluate cost function
Vp = abs(Vtot)+Vtot;
cost =
sort(abs(V1))*sort(abs(V2))'-V1*V2'+0.35*(4*abs(Vtot)*abs(Vtot)'-
Vp*Vp');

end

---------------------------------------------------
filename costfun_graph.m
---------------------------------------------------

function cost = costfun_graph(V1,V2,tau,pha)
%graphs cost function

%apply phase shifts individually to each voltage
V1 = circshift(V1,[0,pha]);
V2 = circshift(V2,[0,-45]);

%generate flipping instruction and apply it to voltage in series
L = length(V1);
flip = vf(tau,L);
Vtot = V1+V2;
Vtot = Vtot.*flip;



% V1 = V1.*flip;
% V2 = V2.*flip;
Vp = abs(Vtot)+Vtot;
cost =
sort(abs(V1))*sort(abs(V2))'-V1*V2'+0.35*(4*abs(Vtot)*abs(Vtot)'-
Vp*Vp');
end
```

----
filename vf.m

```
function flip  = vf(Lp,L)
%generate voltage flips for a given ON/OFF period Lp
%and vector length L
same = -ones(1,floor(Lp));
flip = same;
same = -same;
for i = 1:1:floor(L/Lp)+2
   flip = [flip same];
   same = -same;
end
flip = flip(1:1:L);
end
```
